# Unusual Case of Nephrotic Syndrome From Light Chain Amyloidosis in a 37-Year-Old Patient

**DOI:** 10.7759/cureus.18120

**Published:** 2021-09-20

**Authors:** Omar K Salameh, Matthew C Darok, Jennifer A Kane, Catherine Abendroth, Naman Trivedi

**Affiliations:** 1 Medicine/Nephrology, Penn State Health Milton S. Hershey Medical Center, Hershey, USA; 2 College of Medicine, Penn State College of Medicine, Hershey, USA; 3 Pathology, Penn State Health Milton S. Hershey Medical Center, Hershey, USA

**Keywords:** bleeding disorders, light cast chain nephropathy, plasma cell myeloma, nephrotic syndrome, amyloidosis al

## Abstract

Amyloidosis with renal involvement is a well-known cause of nephrotic syndrome. Immunoglobulin light-chain amyloidosis (AL), which is a result of monoclonal light-chain deposition in the kidney from plasma cell dyscrasia, is rare before the age of 40 and typically occurs in old patients. Most cases of renal amyloidosis in young patients are secondary to chronic inflammatory disease. We are reporting a case of a 37-year-old male who was transferred to our hospital for evaluation of possibly acquired bleeding disorder. He was initially presented to an outside hospital with bleeding per rectum for three days duration and one-week history of abdominal pain and bloating. He was found to have nephrotic range proteinuria with hypoalbuminemia and hyperlipidemia. A kidney biopsy was performed to identify the cause of his nephrotic syndrome, and a biopsy showed AL amyloidosis. Bone marrow biopsy performed showed plasma cell myeloma, and the patient was started on induction chemotherapy.

Even though the incidence of AL amyloidosis is low before age of 40, we should always perform monoclonal gammopathy workup in patients with nephrotic syndrome regardless of the age. Prompt bone marrow biopsy should be performed to confirm the diagnosis, and starting the treatment as one of the factors that affect the prognosis of AL amyloidosis is early diagnosis.

## Introduction

Amyloidosis with renal involvement is a well-known cause of nephrotic range proteinuria. Most cases of renal amyloidosis in younger patients (<40) are due to secondary (AA) amyloidosis, which is amyloidosis secondary to chronic inflammatory disease. In this case, however, workup revealed that the cause of nephrotic syndrome in this 37-year-old male was AL amyloidosis, which is a result of monoclonal light chain deposition in the kidney from plasma cell dyscrasia that typically occurs in much older patients. It is estimated that about 4000 people develop AL amyloidosis each year in the United States. The disease is typically diagnosed between the ages of 50 and 65 years. Although it is rare in people younger than 40 years, people as young as 20 years have also been diagnosed with AL amyloidosis [1]. Several factors affect the prognosis of AL amyloidosis, including early diagnosis, treatment response, and cardiac involvement.

## Case presentation

We report the case of a 37-year-old male with a past medical history of essential hypertension and hyperlipidemia who was transferred to our hospital for further ongoing care for colitis and hematochezia in settings of possible bleeding disorder. The patient was initially presented to another hospital with a one-week history of diffuse abdominal pain, bloating, and a three-day history of bright red blood per rectum. He also reported multiple episodes of vomiting and one episode of gross hematuria. The patient also reported a six-month history of fatigue, generalized body weakness, cold intolerance, swelling in his arms and legs, easy bruising, and unintentional weight loss of 20 pounds over six months duration. In October 2020, he had an episode of profuse gum bleeding after a dental procedure, and he has been currently being worked up by hematology as an outpatient for von Willebrand disease (VWD) versus hemophilia. He had a remote history of proteinuria and was following by nephrology outpatient, but a biopsy was not pursued in the setting of bleeding disorder workup. We were consulted as he was found to have nephrotic range proteinuria with a 24 hr urine protein of 4.61 gm.

On examination, he was afebrile with a blood pressure of 105/64, tachycardia with a heart rate of 112, and normal oxygen saturation on room air. He was in no acute distress. His cardiac and pulmonary exam was unremarkable. Abdominal examination revealed mild diffuse tenderness and dullness on percussion. He had +2 non-pitting edema in the lower extremities. The skin was dry, and no rash was noted. Ultrasound abdomen was done, which showed hepatomegaly with findings of portal hypertension with the slow flow in the main portal veins, recanalized umbilical vein, pericholecystic edema, reversal flow within the splenic vein at pancreas, splenic siderosis, splenic varices, and moderate ascites.

Laboratory workup (Table 1) revealed acute kidney injury (AKI) with serum creatinine of 1.88 mg/dl (baseline creatinine around 1 mg/dl), hyponatremia, hypoalbuminemia, and hyperlipidemia. Hepatitis and human immunodeficiency virus (HIV) serology were negative. Autoimmune workup showed negative antinuclear antibodies (ANA), anti-neutrophil cytoplasmic antibodies (ANCA), and anti-glomerular basement membrane antibody (anti-GBM), normal complement C3, high complement C4, and elevated beta-2 microglobulin. Urine analysis and microscopy showed no white blood cells (WBC) or red blood cells (RBC), random urine protein above 500. 24-hour urine protein showed 4.61-gram proteins, urine protein to creatinine ration of 5.54, and normal serum immunofixation pattern. No monoclonal immunoglobulins were detected. Serum protein electrophoresis (SPEP) report showed increased alpha proteins. No monoclonal immunoglobulins were detected. A urine immunofixation report identifies a free lambda monoclonal immunoglobulin representing less than 10% of total urine protein. The free kappa level was high, and the free lambda level was high; however, the kappa to lambda ratio was low.

**Table 1:** Laboratory workup BUN: blood urea nitrogen; LDL: low-density lipoprotein; HDL: high-density lipoprotein; ANA: antinuclear antibodies; IFA: immunofluorescence assay; HIV: human immunodeficiency virus; HCV: hepatitis C virus; HPF: high-powered field.

Creatinine mg/dl (0.7-1.30)	1.88
BUN mg/dl (6-23)	26
Hemoglobin g/dl (13-17)	14.8
Platelets k/ul (150-350)	580
Albumin g/dl (3.5-5.2)	1.9
Cholesterol mg/dl (normal high < 200)	334
LDL mg/dl (50-130)	293
HDL mg/dl (normal low > 40)	15
Triglycerides mg/dl (normal high < 150)	131
ANA, by IFA	< 1:80, Negative
Complement C3 mg/dl (90-180)	152
Complement C4 mg/dl (10-40)	53
Immunoglobulin G mg/dl (700-1600)	821
Immunoglobulin M mg/dl (40-230)	100
Immunoglobulin A mg/dl (70-400)	497
Immunoglobulin D mg/dl (<= 15.3)	5.6
Beta-2 Micro globulin mg/L (0.80-2.60)	4.50
C-reactive protein mg/dl (<0.50)	0.75
Erythrocyte sedimentation rate mm/hour (0-15)	64
Protein, serum g/dl (6.1-7.9)	5.0
Free Kappa mg/dl (0.33-1.94)	6.53
Free Lambda mg/dl (0.57-2.63)	49.68
Kappa/Lambda Ratio, Serum mg/dl (0.26-1.65)	0.13
M-Spike, Quantitative (24 h)	227.5
HIV	Nonreactive
Hepatitis B surface antigen	Nonreactive
HCV antibody	Nonreactive
Urine hemoglobin	Negative
Urine white blood cells	0-4/HPF
Urine red blood cells	0-4/HPF
24-hour urine protein g/24 hours (normal high < 0.150)	4.611

**Figure 1:**
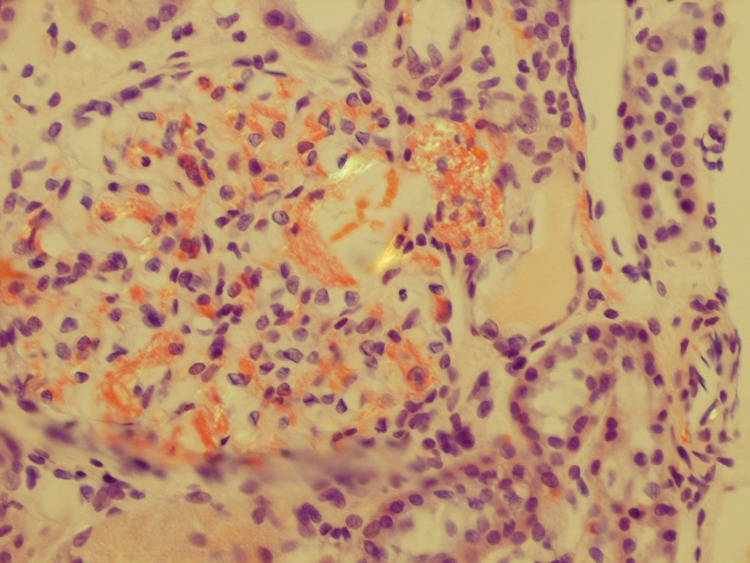
Mesangial deposition of Congo red positive material showing apple-green birefringence under polarized light (CR, x500)

**Figure 2:**
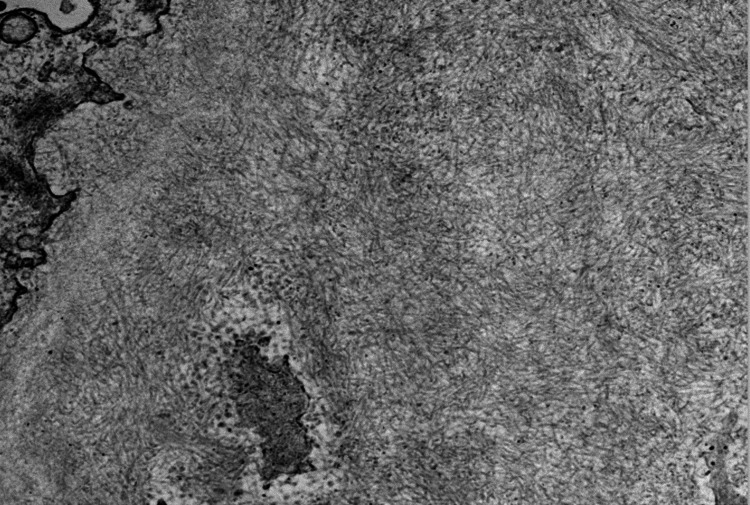
Amyloid fibrils on electron microscopy (x43,800)

A renal biopsy was performed. The tissue process for light microscopy is a single length of renal cortical tissue with a total of 11 glomeruli, four of which are globally sclerotic. The remaining glomeruli revealed a diffuse and global non-nodular increase in eosinophilic extra-cellular mesangial metrical material. The material was weakly PAS positive, silver negative, Congo red positive, and demonstrated apple-green birefringence when examined under polarized light (Figure 1). There were occasional sheaf-like projections perpendicular to the glomerular basement membrane that were highlighted with the Jones silver stain. Tubule demonstrates PAS positive cytoplasmic protein droplets. There is no significant interstitial inflammation. There is mild interstitial fibrosis/tubular atrophy. The mesangial material demonstrated monotypic immunoreactivity for lambda light chains without dominant immunoglobulin expression. Ultrastructural examination confirmed the presence of fine randomly oriented fibrils consistent with amyloid of approximately 10 nm (Figure 2). The kidney biopsy sample was sent to Mayo clinic for liquid chromatography-tandem mass spectrometry (LC-MS/MS): Liquid chromatography-tandem mass spectrometry (LC-MS/MS) detected a peptide profile consistent with AL (lambda)-type amyloid deposition. These findings support the diagnosis of amyloidosis and indicate AL (lambda)-type amyloid deposition.

**Figure 3:**
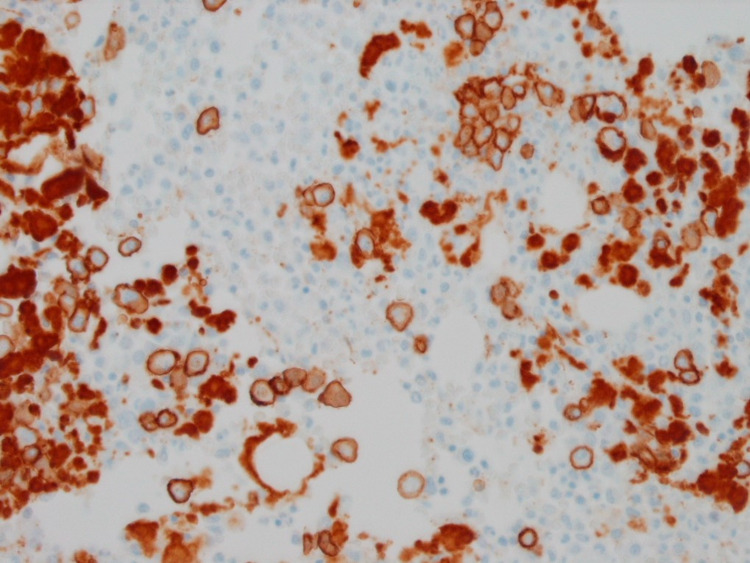
Plasmacytosis in bone marrow (CD138 immunohistochemistry, x500)

Bone marrow biopsy revealed a plasma cell neoplasm, favor plasma cell myeloma (Figure 3). Flow cytometry immunotyping showing CD (cluster differentiation) 19-, CD56-, CD117 +, lambda light chain restricted monotypic plasma cells consistent with plasma cell neoplasm. Echocardiography was done and showed an EF of 70%. The patient was started on CyBorD (Cyclophosphamide, bortezomib and dexamethasone) induction chemotherapy. Workup for bleeding disorders revealed that the patient has Type 2A von Willebrand Disease, likely secondary to the AL amyloidosis.

## Discussion

Amyloidosis with renal involvement is a well-known cause of nephrotic range proteinuria. Most cases of renal amyloidosis in younger patients (<40) are due to AA amyloidosis, which is amyloidosis secondary to chronic inflammatory disease. AL amyloidosis is an uncommon disorder, and the exact incidence is unknown. In the United States, the incidence appears to be stable at approximately 9 to 14 cases per million person-years [2-5]. AL amyloidosis is a disease of older adults. As with other plasma cell dyscrasias, the age-specific incidence rates increase in each decade of life after age 40 years [2]. The median age at diagnosis is 64 years, and less than 5 percent of patients are under the age of 40 [5-10]. The disease is more common in men, with a percentage of 65 to 70 percent of patients are men.

In this case, however, workup revealed that the cause of nephrotic syndrome in this 37-year-old male was AL amyloidosis, which is a result of monoclonal light chain deposition in the kidney from plasma cell dyscrasia that typically occurs in much older patients. The mean onset of AL amyloidosis is in the sixth decade of life. Current literature suggests that less than 1.5% of patients with AL amyloidosis will be diagnosed before the fourth decade of life. Further, as few as 0.64% of patients will be both under 40 and have renal involvement with nephrotic-range proteinuria at the time of diagnosis; in this patient, elevated urine protein was identified months before he was admitted to our hospital, but kidney biopsy and the subsequent diagnosis was delayed since he was undergoing a workup for a new-onset bleeding disorder. He was later found to have Type 2A von Willebrand Disease, likely secondary to the AL amyloidosis [11]. During his inpatient stay and workup, the patient experienced worsening of his abdominal pain and distention, requiring progressively increased diuretic doses and he required paracentesis. The patient was started on CyBorD induction chemotherapy. More prompt investigation and recognition of amyloidosis as a potential cause of nephrotic range proteinuria in this young patient may have reduced the morbidity and complications that were experienced.

Current literature shows multiple case reports of acquired von Willebrand syndrome in patients with AL amyloidosis [11, 12]. However, there are no published studies with population-based evidence to support which clinical factors are related to the development of acquired von Willebrand syndrome in the setting of AL amyloidosis. The mechanism of acquired von Willebrand syndrome in amyloidosis is thought to be similar to that of coagulopathy found in other myeloproliferative and lymphoproliferative disorders, that being due to adsorption of coagulation proteins by the invading amyloid light chains [11]. Importantly, in all published reports, all of the patients presented with severe bleeding diathesis [11, 12]. The diagnosis of acquired von Willebrand syndrome in patients with AL amyloidosis diagnosed at a young age has been reported in three cases before [11]. This similarity adds to the argument of there being an association of acquired von Willebrand syndrome and AL amyloidosis and could support the occurrence in the setting of a young male with bleeding diathesis. More future research is necessary to support these findings statistically. Furthermore, it has been reported that high levels of von Willebrand factor antigen are associated with the prognosis of patients with AL amyloidosis, especially in the setting of endothelial dysfunction and, thus, cardiac dysfunction [13]. Therefore, it is practical to suggest that any patient that presents with severe bleeding diathesis and is found to have AL amyloidosis should be screened for acquired von Willebrand syndrome as diagnosed by reduced von Willebrand factor ristocetin cofactor (VWF: RCo) activity or increased von Willebrand factor antigen (VWF: Ag).

The possibility of AA amyloidosis from an undiagnosed chronic inflammatory disorder was initially considered in this patient since the incidence of AA amyloidosis is higher than the incidence of AL amyloidosis in the third decade of life. The development of nephrotic range proteinuria is associated with chronic inflammatory disorders because AA amyloidosis is a rare but serious complication of sustained inflammation. The most common underlying disorders associated with AA amyloidosis are rheumatoid arthritis, juvenile idiopathic arthritis, inflammatory bowel diseases, spondyloarthritis, psoriasis, and Familial Mediterranean fever; however, over fifty other conditions, including vasculitides, neoplasms, and chronic infections, have also been linked to the development of AA amyloidosis [14, 15]. No single serological test can confirm AA amyloidosis, as the definitive diagnosis is made with tissue biopsy and mass spectrometry, similarly to AL amyloidosis [16, 17]. A thorough history, however, revealed no evidence of an undiagnosed chronic inflammatory disorder in this patient. Erythrocyte sedimentation rate (ESR) and C-reactive protein were elevated upon initial presentation, but these inflammatory markers are non-specific for AA amyloidosis. Additional workup for potential infectious and autoimmune causes, including HIV, hepatitis, and ANA, were all negative.

Hajra and Bandyopadhyay [18] reported a case in 2016 in which a 40-year-old woman presented with spinal deformity and was found to have progressive pedal edema for three months before the presentation. She did not respond to treatment in other hospitals. The patient was found to have nephrotic range proteinuria with hypoalbuminemia. On further investigation, the kappa to lambda ratio was 0.029. Bone marrow biopsy revealed 62% of plasma cells, including binucleated form. Renal biopsy demonstrated lambda light chain deposition and positive birefringence of Congo red-stained material under polarized light. These results led to a diagnosis of AL amyloidosis.

## Conclusions

Even though the incidence of AL amyloidosis is low before age of 40, we should always perform monoclonal gammopathy workup in patients with nephrotic syndrome regardless of the age. In this case, we did a comprehensive workup with SPEP, serum immunofixation, and even urine immunofixation. What was unusual in this case is hepatomegaly with findings of portal hypertension, bleeding diathesis, and early age of onset. Our patient has a history of nephrotic range proteinuria since October 2020. At that time, the biopsy was not pursued in the setting of bleeding disorder workup. This led to a delayed diagnosis of AL amyloidosis. Instead of waiting for a kidney biopsy to be performed, a bone marrow biopsy should be performed to confirm the diagnosis of AL amyloidosis, and starting the treatment as one of the factors that affect the prognosis of AL amyloidosis is early diagnosis.
